# Opposing Calcium-Dependent Effects of GsMTx4 in Acute Lymphoblastic Leukemia: In Vitro Proliferation vs. In Vivo Survival Advantage

**DOI:** 10.3390/ijms26104822

**Published:** 2025-05-18

**Authors:** Souleymane Abdoul-Azize, Rachid Zoubairi, Olivier Boyer

**Affiliations:** UMR 1234, Inserm, Univ Rouen Normandie, 22 Boulevard Gambetta, F-76000 Rouen, France

**Keywords:** ALL, GsMTx4, Ca^2+^ signaling, cell survival

## Abstract

Mechanogated (MG) ion channels play a crucial role in mechano-transduction and immune cell regulation, yet their impact on blood cancers, particularly acute lymphoblastic leukemia (ALL), remains poorly understood. This study investigates the pharmacological effects of GsMTx4, an MG channel inhibitor, in human ALL cells both in vitro and in vivo. Unexpectedly, we found that GsMTx4 remarkably increased basal calcium (Ca^2+^) levels in ALL cells through constitutive Ca^2+^ entry and enhanced store-operated Ca^2^⁺ influx upon thapsigargin stimulation. This increase in basal Ca^2+^ signaling promoted ALL cell viability and proliferation in vitro. Notably, chelating intracellular Ca^2+^ with BAPTA-AM reduces GsMTx4-mediated leukemia cell viability and proliferation. However, in vivo, GsMTx4 decreases cytosolic Ca^2+^ levels in Nalm-6 GFP⁺ cells isolated from mouse blood, effectively countering leukemia progression and significantly extending survival in NSG mice transplanted with leukemia cells (median survival: GsMTx4 vs. control, 37.5 days vs. 29 days, *p* = 0.0414). Our results highlight the different properties of GsMTx4 activity in in vitro and in vivo models. They also emphasize that Ca^2+^ signaling is a key vulnerability in leukemia, where its precise modulation dictates disease progression. Thus, targeting Ca^2+^ channels could offer a novel therapeutic strategy for leukemia by exploiting Ca^2+^ homeostasis.

## 1. Introduction

Approximately one-third of pediatric cancers and three-fourths of leukemias are diagnosed as acute lymphoblastic leukemia (ALL) [1,2,3]. Over time, ALL therapy has been progressively refined through risk stratification, incorporating both clinical and biological criteria. Although 5-year survival rates have significantly improved over the past decades [4], they have recently plateaued. Outcomes for relapsed/refractory ALL remain poor, with survival rates often below 20% in T-ALL and around 50% in B-ALL [5,6]. A deeper understanding of ALL pathogenesis is urgently needed. In this context, we previously identified a critical role for intracellular Ca^2+^ signaling in ALL cell survival [7].

In immune cells, many essential functions are regulated by calcium (Ca^2+^), which can activate or inhibit various physiological and biochemical processes under both normal and pathological conditions. Several studies have reported aberrant Ca^2^⁺ signaling in cancer, which governs key processes such as cell proliferation, invasiveness, angiogenesis, migration, and metastasis [8,9,10].

Mammalian cells ubiquitously express mechanically gated (MG) ion channels [11], which serve as an efficient pathway for Ca^2+^ influx from the extracellular space into the cytosol. The activation of non-selective cation channels by mechanical stretching of the plasma membrane has been demonstrated using patch-clamp techniques in various tissues, including neurons, muscles, epithelial cells, and osteoblasts [12]. However, the role of Ca^2+^-permeable MG channels in blood cells, particularly in ALL, remains poorly understood.

Growing evidence suggests that, alongside biochemical signals, mechanotransduction cues play a crucial role in regulating immune cell functions. Key mechanosensors, such as the PIEZO family of mechanically activated cation channels, convert mechanical stimuli into intracellular signals, leading to Ca^2^⁺ fluxes, cytoskeletal remodeling, and transcriptional regulation [13]. Consequently, an increasing number of small molecules that selectively inhibit the PIEZO channel are under development.

GsMTx4 is a peptide isolated from tarantula venom that inhibits MG channel activation [14] with cell type-dependent effects. For instance, GsMTx4 has been reported to prevent neuronal damage and protect against lysophosphatidylcholine-induced astrocyte toxicity in vivo [15]. Additionally, it has been shown to reduce TRAIL-mediated cancer cell cytotoxicity [16], suppress neurogenesis while enhancing astrogenesis in human neural stem cells [17], and inhibit leptin-induced MLC-2 and AMPK phosphorylation in breast epithelial cells [18]. However, its impact on ALL cell functions remains unexplored.

To address this gap, we evaluated the pharmacological properties of GsMTx4 in ALL cells. Using five different ALL cell lines, we examined its effects on ALL cell proliferation, viability, and Ca^2+^ signaling in vitro, as well as overall survival in NSG mice in vivo. Our results show that GsMTx4 enhances ALL cell survival in vitro while prolonging overall survival in mice, an effect associated with the modulation of Ca^2+^ signaling in ALL cells.

## 2. Results

### 2.1. MG Ion-Channel Inhibitor GsMTx4 Enhances Basal Ca^2+^ Levels in ALL Cell Lines

GsMTx4 was developed as a highly selective inhibitor of mechanically induced Ca^2+^ signaling through MG channels [19,20]. However, published data on its direct effects on cells are limited, and even scarcer in immune cells. While exploring how GsMTx4 could modulate Ca^2+^ signaling in ALL cells, we initially hypothesized that GsMTx4 would inhibit Ca^2+^ signaling in these leukemia cells.

To address this question, we first investigated whether varying concentrations of the compound, within a range of non-cytotoxic doses known to inhibit MG channel activity in various vertebrate cell types [21], would impact the basal cytosolic Ca^2+^ level in ALL cells. We monitored cytosolic Ca^2+^ concentration with the Fura-2 QBT probe in five different ALL cell lines (two B-ALL and three T-ALL cell lines) pre-treated with either the vehicle or the MG channel blocker GsMTx4.

**Figure 1:**
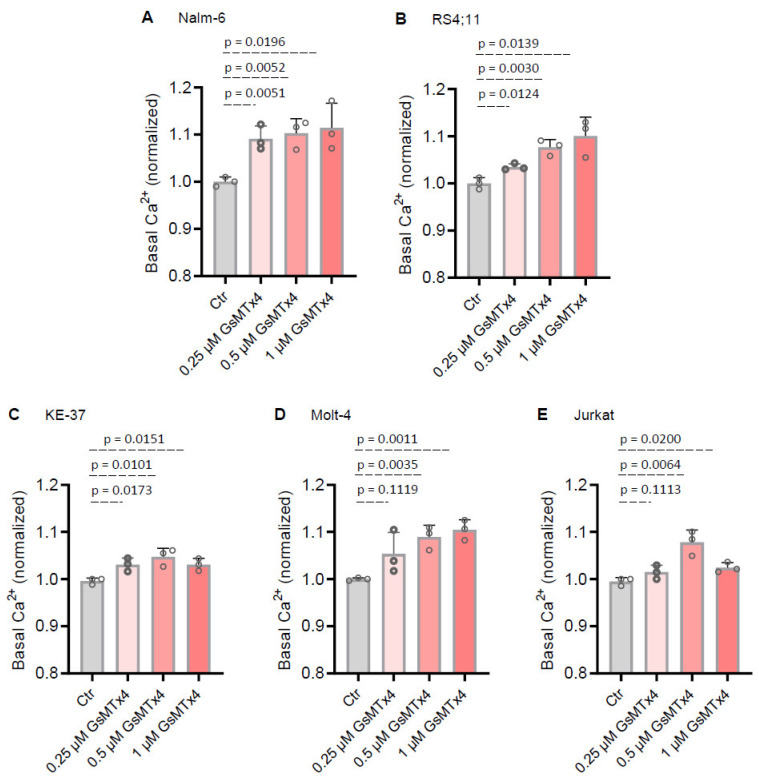
Mechanogated ion channel inhibitor (GsMTx4) enhances basal Ca^2+^ levels in ALL cell lines. The basal Ca^2+^ level was used as a read-out to measure the impact of GsMTX4 on constitutive Ca^2+^ channel activity in ALL cells. (**A**–**E**) ALL cell lines pre-treated with control (Ctr, DMSO) and GsMTx4 using the ratiometric Ca^2+^ indicator Fura-2 QBT. The basal Ca^2+^ level was evaluated before normalization as the average of initial F_340_ nm/F_380_ nm values. Data are mean ± SEM (*n* = 3). Statistical significance was analyzed by two-tailed, unpaired Student’s *t*-tests.

Surprisingly, the basal Ca^2+^ level in GsMTx4-treated ALL cells was higher compared to vehicle-treated ALL cells (Ctr) (Figure 1). Furthermore, this increase in basal Ca^2+^ levels in ALL cells was dose-dependent (Figure 1). Taken together, these results suggest that the effect of GsMTx4 on ALL cells is characterized by enhanced basal Ca^2+^ signaling, likely supported by increased activity of membrane Ca^2+^ channels.

### 2.2. GsMTx4 Increases Cytosolic Ca^2+^ Levels Through Activation of Constitutive Ca^2+^ Entry in ALL Cell Lines

To gain a better understanding of these unexpected effects on basal Ca^2+^ signaling and considering that ALL cells are non-excitable, we hypothesized that GsMTx4 could modulate membrane Ca^2+^ channel activity. Indeed, we recently described that basal Ca^2+^ signaling in leukemia cells is strongly influenced by the constitutive activity of Ca^2+^ channels [22].

**Figure 2:**
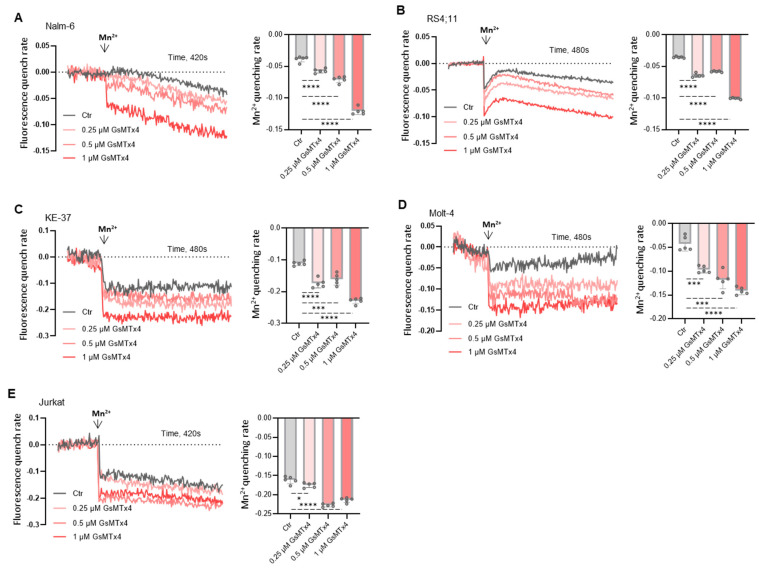
GsMTx4 increases Mn^2+^ influx in ALL cell lines. The quenching of Fura-2 fluorescence by Mn^2+^ in ALL cell lines was measured at 360 nm (the Ca^2+^-independent excitation wavelength of Fura-2). (**A**–**E**) ALL cells before exposure to Mn^2+^ (10 μM) were preincubated during Fura-2 GBT loading with GsMTx4. Results express normalized fluorescence and quenching peak quantification. Data are mean ± SEM (*n* = 5). Statistical significance was analyzed by two-tailed, unpaired Student’s *t*-tests. * *p* < 0.05, *** *p* < 0.001, and **** *p* < 0.0001.

As a first approach, we evaluated the effect of GsMTx4 on constitutive Ca^2+^ entry in ALL cells by using the experimental method Mn^2+^ quenching assay. The results from this analysis (Figure 2) revealed that the MG channel blocker stimulated constitutive Ca^2+^ entry in a dose-dependent manner compared to the Ctr in these five ALL cell lines.

**Figure 3:**
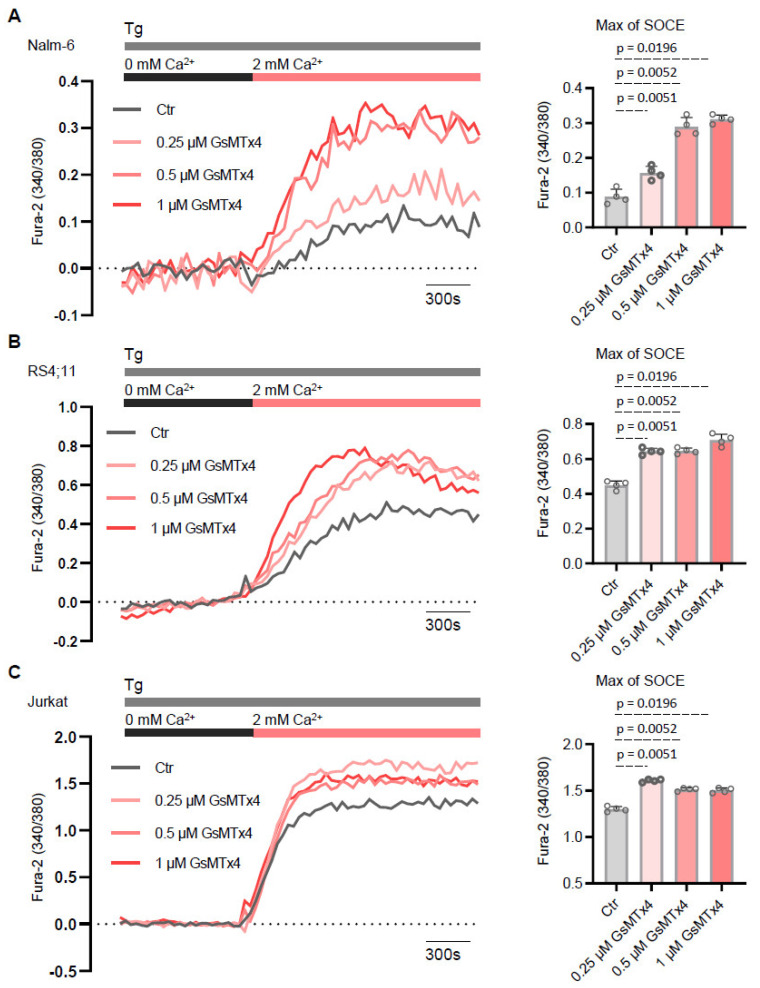
GsMTx4 potentiates store-operated Ca^2+^ entry (SOCE) in ALL cell lines. (**A**–**C**) Representative traces and quantification of SOCE peak upon store depletion in 0 mM Ca^2+^ with 2 µM TG (thapsigargin) followed by addition of 1.8 mM Ca^2+^ to the solution. Cells before exposure to TG in Ca^2+^ buffer were preincubated during Fura-2 QBT loading with GsMTx4. Data are mean ± SEM (*n* = 4). Statistical significance was analyzed by two-tailed, unpaired Student’s *t*-tests.

As a second approach, we evaluate whether GsMTx4 potentiates Ca^2+^ entry across the plasma membrane through store-operated Ca^2+^ entry (SOCE). This pathway is a widely conserved mechanism in non-excitable cells like ALL cells [23] and is triggered by the depletion of Ca^2+^ stores within the endoplasmic reticulum (ER). To test the effects of GsMTx4 in ALL cell lines, we measured Ca^2+^ influx following ER store depletion with thapsigargin (Tg, 1 μM), a pharmacological inhibitor of the sarco–endoplasmic reticulum Ca^2+^-adenosine triphosphatase (SERCA). GsMTx4-treated ALL cells and Ctr ALL cells were initially stimulated with Tg in a nominally Ca^2+^-free solution. The subsequent addition of 2 mM extracellular Ca^2+^ in the bath solution revealed the activation of the Ca^2+^ entry phase across the plasma membrane, mediated by the SOCE pathway (Figure 3). Consistent with the results showing potentiation of constitutive entry of Ca^2+^, GsMTx4 significantly enhanced SOCE in a dose-dependent manner compared to Ctr ALL cells.

These findings indicate that the MG channel inhibitor interacts with a Ca^2+^-sensitive membrane channel and suggest that GsMTx4 contributes significantly to basal Ca^2+^ by modulating constitutive Ca^2+^ activity in resting ALL cells.

### 2.3. GsMTx4-Mediated Intracellular Ca^2+^ Signaling Positively Regulates ALL Cell Proliferation and Viability

**Figure 4:**
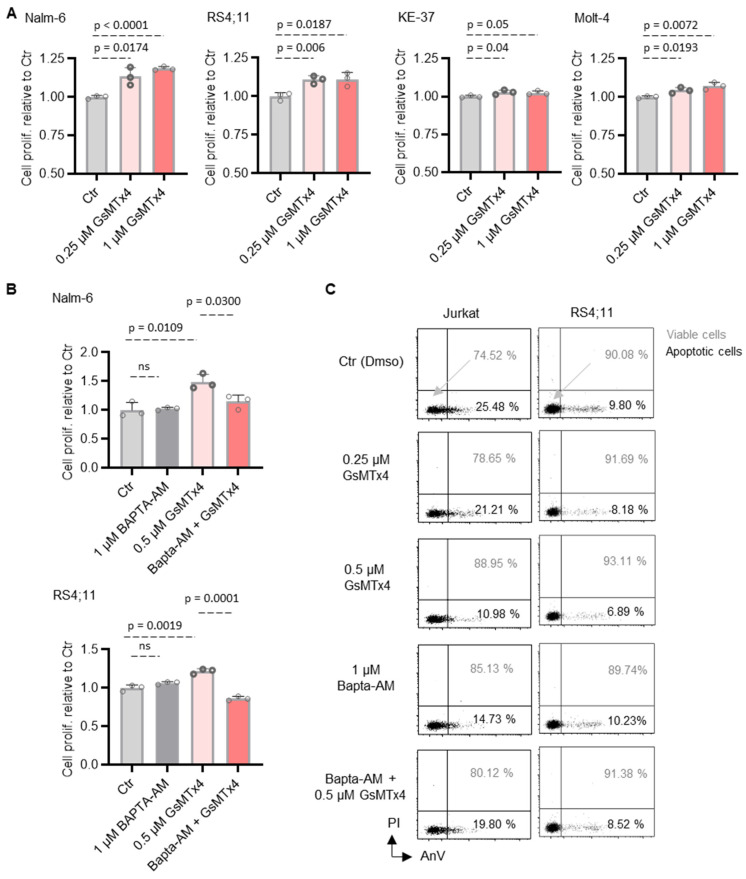
GsMTx4 stimulates proliferation and viability of ALL cells through Ca^2+^ signaling. (**A**) ALL cell lines were treated with GsMTx4 for 48 h. Then, the proliferation rate was determined by CCk-8 staining. The percentage was established after normalizing on control (Ctr) cells. (**B**,**C**) ALL cell lines were treated with GsMTx4 for 48 h in the absence or presence of BAPTA-AM (1 μM). (**B**) Cell proliferation rate was determined by CCK-8 assay. (**C**) Cell viability was determined by flow cytometry after annexin V/PI staining. Viable cells represent the percentage of annexin V-FITC/PI- negative population. Data are mean ± SEM (*n* = 3). Statistical significance was analyzed by two-tailed, unpaired Student’s *t*-tests; ns, not significant.

To assess whether GsMTx4 treatment could have functional consequences on ALL cell survival and proliferation, we first assessed cell proliferation using the CCK-8 assay. We found that the proliferation of ALL cell lines was significantly increased after 48 h of treatment with GsMTx4 compared to the Ctr in a dose-dependent manner (Figure 4A). Next, since GsMTx4 enhanced intracellular Ca^2+^ levels, we then examined the role of this cytosolic Ca^2+^ in GsMTx4-induced ALL cell proliferation. To this end, ALL cells were pre-incubated with or without Bapta-AM (1 μM), an intracellular Ca^2+^ chelator, and then exposed or not to GsMTx4. Notably, Bapta-AM (1 μM) alone had no significant impact on ALL cell proliferation (Figure 4B), whereas, in the presence of Bapta-AM, GsMTx4 failed to stimulate ALL cell proliferation compared to GsMTx4 alone (Figure 4B).

Secondly, the viability of two ALL cell lines was analyzed by flow cytometry using the AnnexinV/PI assay. The viability rate of ALL cells increased progressively with increasing exposure concentrations of GsMTx4, corresponding to a decrease in the rate of apoptotic cells (Figure 4C). Similarly to the proliferation assay, and as expected, BAPTA-AM obviously attenuated the GsMTx4-induced increase in ALL cell viability (Figure 4C), providing a potential mechanism by which GsMTx4 potentiates Ca^2+^ influx and enhances ALL cell function in vitro.

### 2.4. GsMTx4 Prolongs Overall Survival of NSG Mice by Decreasing Cytosolic Ca^2+^ Levels of ALL Cells In Vivo

**Figure 5:**
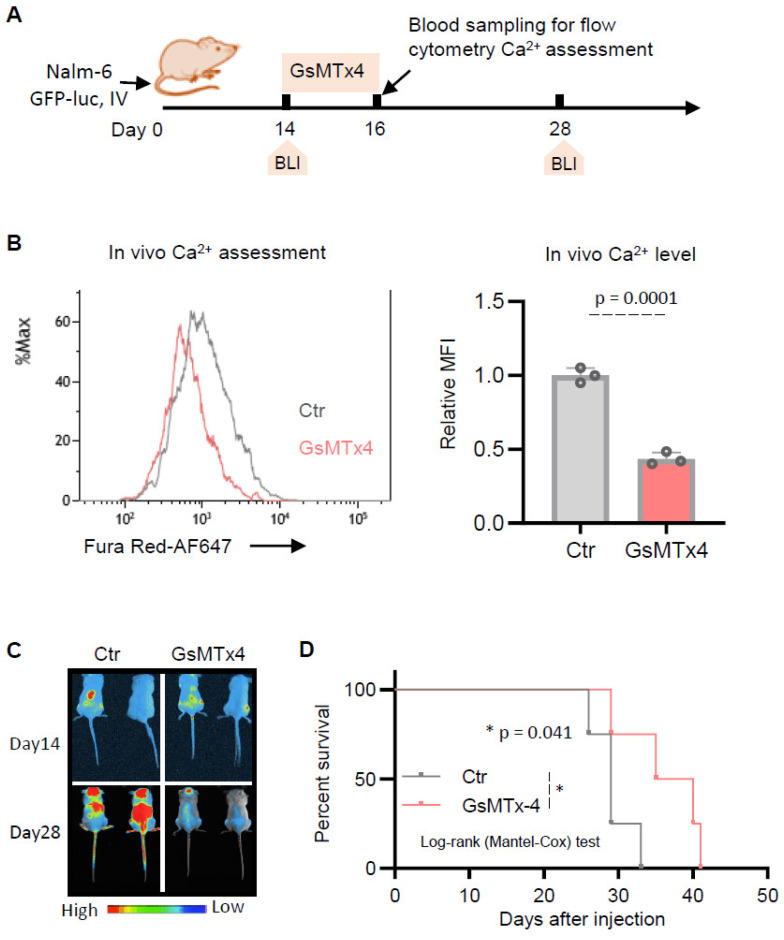
GsMTx4 decreases cytosolic Ca^2+^ levels of ALL cells and prolongs overall survival of NSG mice in vivo. (**A**) Experimental design of NSG mice treatment experiments. BLI, bioluminescence imaging. (**B**) Flow cytometry Ca^2+^ assessment in Nalm-6 GFP+ cells in the blood at day 16. (**C**) An example of bioluminescence imaging of Nalm-6 GFP/luc at days 14 and 28 of tumor challenge. (**D**) Kaplan–Meier survival analysis of mice transplanted with Nalm-6 GFP-luc cells and treated with Ctr (DMSO, *n* = 4) and GsMTx4 (*n* = 4). Statistical significance was calculated with the log-rank (Mantel–Cox) test, (* *p* = 0.041).

We next investigated the effect of GsMTx4 in vivo. To do so, Nalm6-luciferase-GFP cells were injected into immunodeficient NSG mice, followed by intraperitoneal administration of either DMSO (Ctr) or GsMTx4 (Figure 5A).

At the end of treatment, Nalm-6 GFP^+^ cells were collected from the blood of NSG mice on day 16 (Figure 5A) in both groups to measure intracellular Ca^2+^ levels in vivo using flow cytometry. In contrast to its in vitro effects, we observed that cytosolic Ca^2+^ levels were reduced following GsMTx4 treatment compared to the Ctr (Figure 5B). This decrease was associated with a reduction in tumor burden, as measured by bioluminescence imaging (Figure 5C), and an overall prolongation of mouse survival (Figure 5D). Taken together, these data support a model in which MG channel inhibitors could have potential in vivo benefit as a therapeutic target for ALL.

## 3. Discussion

Recent studies have begun to uncover the role of PIEZO channels in immune cell regulation [13], but data on selective inhibitors like GsMTx4 remain limited. Here, we characterized the in vitro and in vivo impact of GsMTx4 on ALL (both B- and T-ALL) cells. Given the fundamental role of Ca^2+^ signaling in leukemia progression, we focused specifically on this pathway as a key regulator of cell survival and proliferation. The goal of this study was not to identify downstream transcriptional or signaling pathways but to investigate how GsMTx4 modulates Ca^2+^ dynamics in distinct biological contexts.

In vitro, GsMTx4 promoted ALL cell viability and proliferation by increasing basal cytosolic Ca^2+^ levels. This effect appeared to be mediated by constitutive Ca^2+^ entry and an enhancement in store-operated Ca^2+^ entry, as evidenced by the potentiation of thapsigargin-induced Ca^2+^ influx. Interestingly, this finding suggests that its effect on Ca^2+^ signaling is not limited to the inhibition of MG channels like Piezo1 but may also involve indirect modulation of the STIM1/Orai1 pathway. Given that SOCE is tightly regulated by membrane architecture and ER-plasma membrane contact sites, it is plausible that GsMTx4, through its amphipathic nature and interaction with the lipid bilayer [24], alters local membrane tension or protein dynamics in a way that facilitates STIM1 activation or Orai1 recruitment. This mechanism could explain the unexpected rise in intracellular calcium levels observed in our study. The reduction in viability and proliferation upon BAPTA-AM treatment confirmed the Ca^2+^ dependence of this effect. These findings align with reports showing that GsMTx4 increased QGP-1 cell viability and 5-hydroxytryptamine (5-HT) levels [25], with the secretion of 5-HT linked to elevated intracellular Ca^2+^ signaling [26]. Additionally, previous studies indicated that genetic deletion or inhibition of PIEZO1 promoted the expansion of T cell populations [27] or protected chondrocytes from apoptosis under mechanical strain [28]. In contrast, partial knockdown of PIEZO1 in human CD4⁺ T cells impaired proliferation by disrupting calpain-dependent immunological synapse stabilization [29].

In vivo, however, GsMTx4 significantly extended survival in NSG mice xenografted with leukemia cells, suggesting strong potential for clinical applications. Analysis of GFP⁺ Nalm-6 cells isolated from mouse blood revealed a reduction in cytosolic Ca^2+^ levels in GsMTx4-treated ALL cells. This suggests that while GsMTx4 promoted Ca^2+^ influx in vitro, it disrupted leukemia cell Ca^2+^ homeostasis in vivo, potentially due to microenvironmental factors such as extracellular matrix stiffness, cytokine availability, or mechanical forces [30], which could alter MG channel activity and Ca^2+^ flux in leukemic cells. Indeed, similar discrepancies between in vitro and in vivo effects have been observed in mechanotransduction pathways, where cell behavior is influenced by dynamic interactions with the extracellular niche [30]. These apparently contradictory findings may be explained by the fact that GsMTx4 modulates various mechanosensitive channels beyond PIEZO, either by inhibition or potentiation [31]. Furthermore, GsMTx4 may impact other mechanosensitive proteins, such as mechanoenzymes and GPCRs [31], which contribute to Ca^2+^ signaling regulation. The reduced cytosolic Ca^2+^ observed in ALL cells isolated from GsMTx4-treated mice might indicate a disrupted Ca^2+^ homeostasis that hinders leukemia cell survival in the bone marrow niche. Consistently, in an osteoarthritis (OA) rat model, GsMTx4 alleviated OA by reducing intracellular Ca^2+^ concentration [28]. Additional beneficial in vivo effects of GsMTx4 have been reported. In mice with chronic pulmonary hypertension (PH), treatment with GsMTx4 results in the regression of pulmonary vascular remodeling and a partial reversal of established PH [32]. Furthermore, GsMTx4 has been shown to mitigate histological abnormalities and inflammatory responses in ventilator-induced lung injury, significantly reducing mortality in a rat model [33].

These findings underscore Ca^2+^ signaling as a crucial therapeutic target in leukemia, where its modulation can either promote leukemia cell survival or, conversely, create an unfavorable environment for disease progression. From a therapeutic perspective, our study raises intriguing questions about the role of MG channels in hematologic malignancies. While MG channels are well characterized in mechanically active tissues, their role in hematologic malignancies remains underexplored. Future studies should investigate their potential as therapeutic targets, particularly in combination with existing Ca^2+^-modulating or immune-based therapies. Understanding the differential effects of GsMTx4 in vitro and in vivo could provide new strategies to selectively disrupt leukemic Ca^2+^ signaling while preserving normal hematopoiesis.

## 4. Materials and Methods

### 4.1. Cell Lines, Cell Culture, and Reagents

The human T-ALL cell line KE-37 (DSMZ^®^, Braunschweig, Germany), MOLT-4 (DSMZ^®^, Braunschweig, Germany), Jurkat (ATCC^®^, Manassas, VA, USA) and the human B-ALL cell line Nalm-6 (DSMZ^®^, Braunschweig, Germany) and RS4;11 (DSMZ^®^, Braunschweig, Germany) were used in this study. All ALL cells were cultured in complete RPMI 1640 medium containing 10% fetal bovine serum and antibiotics (penicillin and streptomycin) (Sigma^®^, Saint-Quentin-Fallavier, France) and maintained at 37 °C in a 5% CO_2_ humidified atmosphere. Fura-2/QBT was purchased from Molecular Devices, Winnersh, UK. Thapsigargin, dimethylsulfoxide (DMSO), and manganese were obtained from Sigma^®^, France. GsMTx4 and Bapta-AM were obtained from Abcam, Cambridge, UK.

### 4.2. Animal Experiments

NSG mice (central animal facility of the University of Rouen) were used at 10 to 12 weeks old. NSG mice injected intravenously with Nalm-6 GFP/luciferase cells were treated after engraftment was established (luminescent flux assessed by bioluminescence imaging). This leukemic cell line was selected for in vivo studies due to its well-established capacity to engraft and proliferate in immunodeficient mouse models, as well as its compatibility with in vitro functional assays. Mice were randomized into two groups to equally distribute the leukemic burden (as assessed by bioluminescence). GsMTx4 (dissolved in PBS) was administered intraperitoneally (100 μL per mouse) at a concentration of 80 µg/kg for 3 days. The endpoint for leukemic-free survival was reached when mice had their hind limbs paralyzed.

### 4.3. Intracellular Ca^2+^ Measurement

ALL cells were loaded with Fura-2 QBT (Molecular Devices, R8198, Winnersh, UK) for 60 min at 37 °C in buffer containing (in mM) 5 KCl, 135 NaCl, 1 MgCl_2_, 2–4 CaCl_2_, 10 HEPES, 10 glucose, pH 7.4, in the presence or absence of GsMTx4 on Cell-Tak (Corning, NY, USA) precoated 96-well plates. After 100 s of recording, cells were stimulated with test compounds. Store depletion was induced by stimulating the cells with 2 μM thapsigargin (Invitrogen, Carlsbad, CA, USA) in Ca^2+^-free buffer, and Ca^2+^ influx was induced after adding 2 or 4 mM Ca^2+^ buffer to the cells at 750 s. Cytosolic and SOCE Ca^2+^ entry were quantified by the peak of the F340/F380 ratio. For experiments in Ca^2+^-free buffer, CaCl_2_ was replaced by EGTA (2 mM). Changes in Fura-2 ratios (F340/380) were measured using a FlexStation 3 Multi-Mode Microplate Reader (Molecular Devices) and analyzed using the SoftMax Pro 7.1 software (Molecular Devices).

### 4.4. Basal Cytosolic Ca^2+^ Measurements

Basal Ca^2+^ levels were monitored with the cytosolic Ca^2+^ indicator Fura-2 QBT. Cells were loaded for 60 min with 2 µM Fura-2 QBT at 37 °C in Ca^2+^ buffer solution (in mM, 5 KCl, 135 NaCl, 1 MgCl_2_, 10 HEPES, 1 Na_2_HPO_4_, pH 7.4) and 10 mM glucose and 1.8 mM CaCl_2_, in the presence or absence of GsMTx4. Fluorescence was monitored on a FlexStation 3 multi-mode microplate reader by alternately exciting the Ca^2+^ indicator at 340 and 380 nm and collecting emitted fluorescence at 510 nm. Basal Ca^2+^ levels were estimated as the average of initial F_340_ nm/F_380_ nm values.

### 4.5. Mn^2+^ Quenching

For measurements of Mn^2+^ quenching, ALL cells loaded with Fura-2 QBT were transferred to Ca^2+^-free solution and stimulated with 10 µM Mn^2+^, and fluorescence was measured at an emission wavelength of 360 nm before and after Mn^2+^ addition.

### 4.6. CCK-8 Cell Proliferation Assay

Cell Counting Kit-8 (CCK-8) assay (Sigma, France) was used to assess cell proliferation according to the instructions. Briefly, cultured cells were seeded in 96-well culture plates with or without GsMTx4. After 48 h, 10 μL of reagent solution was added to each well, and the plates were incubated at 37 °C for 3 h. Absorbance was read at 450 nm under Flexstation-3 Molecular Devices (Winnersh, UK).

### 4.7. Bioluminescent Imaging

NSG mice were imaged with the Vilber Smart In Vivo Imaging System (Vilber Lourmat, Marne-la-Vallée, France). Mice were sedated with 2.5% isoflurane, followed by subcutaneous XenoLight D-Luciferin-K+Salt Bioluminescent Substrate (Elmer Perkin, Waltham, MA, USA) injection and subsequent imaging. Quantification and image processing were performed using Newton 7.0 Software (Vilber Smart Imaging, Marne-la-Vallée, France).

### 4.8. Flow Cytometry Ca^2+^ Measurement

Cytosolic Ca^2+^ levels were monitored with cytosolic Ca^2+^ indicator Fura Red™, AM. ALL cells were loaded for 30 min with 2 µM Fura Red at room temperature in the buffer described above, followed by a de-esterification step of 30 min in the absence of probes. Flow cytometry data were collected on a BD LSRFortessa and analyzed with Kaluza software.

### 4.9. Apoptosis Assay

B-ALL cells were treated for 48 h in the presence or absence of GsMTx4. The apoptotic rate was assessed by annexin V-FITC/PI staining (BioLegend, Paris, France). Annexin V-FITC- and propidium iodide-positive cells were determined by flow cytometry using a LSR-Fortessa (BD Biosciences, Le Pont de Claix, France) flow cytometry. All flow cytometry data were analyzed using Kaluza 1.5 software.

### 4.10. Statistical Analysis

Statistical analyses were performed using the PRISM Software 8.0.2 (GraphPad Software, (La Jolla, CA, USA) using unpaired *t*-tests. Data are expressed as mean ± standard error of the mean (SEM) values, and *p* < 0.05 was considered statistically significant.

## Data Availability

The analyzed data and original contributions in this study are included in the article. Further inquiries can be directed to the corresponding author.
